# Patient perspectives on the role of nurses in HIV pre-exposure prophylaxis care (PrEP-RN)

**DOI:** 10.1371/journal.pone.0288283

**Published:** 2023-07-19

**Authors:** Lauren Orser, Patrick O’Byrne, Dave Holmes

**Affiliations:** School of Nursing, University of Ottawa, Ottawa, Ontario, Canada; Shahid Beheshti University of Medical Sciences School of Dentistry, ISLAMIC REPUBLIC OF IRAN

## Abstract

**Introduction:**

In response to ongoing new HIV diagnoses among gay, bisexual, and other men who have sex with men (gbMSM) and limited access points for HIV pre-exposure prophylaxis (PrEP) care, we established Canada’s first nurse-led HIV prevention service in Ottawa, Canada—PrEP-RN. As part of this service, registered nurses became the primary provider in PrEP delivery and monitoring.

**Objectives:**

To (1) gather patients’ sentiments and experiences related to nurse-led PrEP and (2) identify the implications for nurses working in sexual healthcare.

**Methodology:**

Qualitative interviews were conducted with 14 gbMSM participants who had received, or were presently enrolled in, HIV prevention care from nurses in the PrEP-RN clinic. Interview transcripts were reviewed and analyzed using thematic analysis.

**Results:**

Our analysis revealed two major themes of: *The Sexual Health Nurse as the Expert* and *Patients Reliance on Nurses*. The first theme discussed patients’ positive attitudes toward nurses, in terms of the knowledge nurses possessed and the kind and efficient services they. The accommodating nature of nurses, however, led patients to become dependent on their care, which was the focus of the second theme. This reliance on nurses created challenges when patients transitioned from PrEP-RN to alternate providers for ongoing care.

**Conclusion:**

These findings were examined to understand the effect of patients’ perceptions of nurses on nursing practice. Despite patients’ confidence in nurses’ ability to provide PrEP care, the expectations they placed onto nurses to address the totality of their needs created competing demands for nurses to be both a leader in HIV prevention care—and fulfill the image of the caring, healthcare ‘hero’, which created feelings of moral distress among nurses. As increasing initiatives focus on task-shifting of healthcare roles to nurses, understanding the patients’ perspective is essential in maintaining effective nurse-patient relationships.

## Background

Throughout Canada, HIV infections continue to occur, most notably, among persons who identify as gay, bisexual, or men who have sex with men (gbMSM) [1]. gbMSM were noted to account for 43.8% of all HIV diagnoses in Canada in 2020 [1]. Presently, the estimated annual HIV incidence rate for sexually active gbMSM in Canada is 166.2/100,000 –indicating that sexual transmission of HIV remains an ongoing concern for this group [1]. Elevated rates of new HIV infections among gbMSM have also been observed in the United States [2], United Kingdom [3], and Australia [4] (as countries that share similar population characteristics and healthcare systems to Canada). Data current to 2019 show HIV diagnosis rates among gbMSM as follows: in the United States– 70% [2], in the United Kingdom– 41% [3], and in Australia– 47% [4]. In Ottawa, Canada (where this research occurred), gbMSM historically accounted for the highest proportion of new HIV diagnoses [5], with 77% being recently acquired infections in Canadian-born, white, gay men who were an average age of 36 years old.

Ongoing HIV transmission among gbMSM can pose increased pressures for nurses working with gbMSM patients, particularly those in the specialized domains of public health and sexual health nursing. Some such stressors can relate to nurses’ observation of behavioural risk practices among gbMSM which can contribute to HIV vulnerabilities and the previously limited resources available for nurses to provide to gbMSM or reported barriers of such patients to accessing and engaging in HIV prevention care [6].

Over the past ten years in Canada, novel approaches to HIV prevention emerged aimed at curbing the rate of HIV infections among at-risk groups, such HIV chemoprophylaxis (pre- and post-exposure medications) and peer and self-testing initiatives [7–9]. The implementation of these strategies provided more tangible solutions to HIV prevention than strict risk counselling, which has been shown to be ineffective at reducing rates of sexually transmitted infections and HIV among persons seeking rapid HIV testing in a clinical setting [10]. One chemoprophylaxis strategy nurses can offer to patients is HIV pre-exposure prophylaxis, or PrEP, which is available in oral or injectable forms and is reported to prevent HIV acquisition by upwards of 99% [7]. Given its efficacy, it is recommended that nurses offer PrEP referrals to gbMSM with objective indicators for HIV acquisition—such as of the diagnosis of a bacterial sexually transmitted infection (STI) or following a known or potential sexual exposure to a person with transmissible HIV [6, 7]. The advent of this method of HIV chemoprophylaxis and active-offer referrals by public health and sexual health nurses to higher-risk patients has led to a coincident reduction in new HIV diagnoses among gbMSM in some Canadian cities; one being Ottawa, Canada, where this research occurred [11].

The widespread implementation of PrEP services aimed at gbMSM also corresponded with the shifting of HIV prevention services from physicians to nurses to accommodate the growing demand for PrEP and limited points for clinical access. In particular, primary care physicians felt they lacked the knowledge and skill to provide ‘specialist services’ (in this case, the administration of antiretroviral medications for HIV prevention) [12]. However, because PrEP care is generally provided according to a specified set of guidelines, to address this health systems barrier to PrEP access, we created a nurse-led PrEP service (entitled PrEP-RN), which followed the Canadian guidelines [7] and recommended that patients who used daily oral PrEP completed testing for HIV, STIs, and kidney function every 3 months and received counselling related to HIV and STI risk and PrEP medication use at that same time. As part of Canada’s first wholly nurse-led PrEP service [13], we established protocols for registered nurses to complete PrEP assessments, follow-up, and care within our local sexual health clinic. Since the establishment of PrEP-RN, several other nurse-led PrEP models [14, 15] have been established throughout Canada, which has led to an increase in PrEP access points for gbMSM seeking HIV prevention care.

While a great deal of literature exists related to the perceptions and experiences of PrEP use among gbMSM, less is known about the expectations of this group related to PrEP care—particularly when this care is delivered by nurses—and the effect of these perspectives on the role of nurses in HIV prevention care. Using data from qualitative interviews completed with 14 gbMSM patients who accessed PrEP care through PrEP-RN, we were able to obtain new insights on nurse-led HIV prevention services. Specifically, our objectives for this analysis were to (1) gather patients’ sentiments and experiences related to nurse-led PrEP and (2) identify the implications for nurses working in specialized domains of healthcare, such as sexual and public health.

## Methodology

### PrEP-RN study

The PrEP-RN clinic was implemented in August 2018 as a research study at the University of Ottawa. The overarching aims of this study were to explore the feasibility of registered nurses completing care (as opposed to physicians) and to provide rapid access to HIV prevention care for individuals identified as being at elevated risk for HIV acquisition during public health follow-up for STIs or sexual health visits. Qualifying patients for PrEP-RN were those who met the following criteria [13]: diagnosis of rectal chlamydia, rectal gonorrhea, lymphogranuloma venereum, and/or infectious syphilis, sexual and/or needle-sharing contacts of a partner with transmissible HIV, use of HIV post-exposure prophylaxis (PEP), or those considered to at-risk for HIV based on clinical judgement. Recruitment and clinical services occurred at the Sexual Health Clinic in Ottawa in collaboration with the University of Ottawa, School of Nursing.

Patients who agreed to a referral for PrEP care were offered the option to access services either at our nurse-led clinic or with a local infectious disease provider. Those who elected to participate in PrEP-RN received care according to the Canadian PrEP guidelines [7]. As part of this follow-up, registered nurses operating under medical directives from nurse practitioners, completed intake assessments, provided clinical services (e.g., STI screening, vaccination, and treatments), monitored tests results, and dispensed prescriptions for PrEP. All PrEP-RN patients were enrolled for a period of up to one-year. After this point, they were referred to an alternate healthcare provider for ongoing care—with the intention of building the capacity of primary care and community providers to prescribe PrEP. Study funds were used to provide oral PrEP medications at a reduced cost for the first 3 months to participants who did not have access to medication insurance.

### Design

For the qualitative evaluation component of this study, we applied a pragmatic theoretical lens. This approach focuses on practical reality (based on the views and experiences of individuals), rather than a single, objective view of reality [16]. That is, reality is centered in the individual actions and settings of participants and is constantly evolving based on time, place, and experience. The pragmatic approach is useful in this research, as it aims to help “solve practical problems” [16, p.2]. Because little information exists on nurse-led PrEP, a practical lens is useful to help inform future iterations of services that are based on the needs and experiences of individuals who are accessing this type of care. In other words, the study was designed in such a way that the lived realities of participants remained central elements in the development, analysis, and interpretation of the study findings.

### Sampling

Qualitative interviews for the PrEP-RN study were completed from June-September 2020. These interviews were carried out by a research assistant who has no training or designation as a registered nurse and was not involved in nurse-led PrEP delivery. A total of 35 individuals who had indicated their consent on the written research consent form (in the PrEP-RN clinic) to be contacted for an interview were approached through an e-mail invitation; 14 agreed to participate.

### Data collection

Due to the COVID19 pandemic lockdowns, interviews were conducted online by a research assistant. Interviews were conducted once and ranged in duration from 35–60 minutes. These interviews were video recorded and transcribed verbatim by LO (author). Interview prompts relating to participants’ experiences with accessing PrEP-RN, receiving PrEP care from nurses, and recommendations for program improvement were developed in advance of the interviews to guide discussions (See Table 1). The semi-structured nature of the interviews also allowed time for participants to raise items of importance to them related to their experience accessing nurse-led PrEP. Participant characteristics were obtained from a sociodemographic survey conducted at the time initial enrollment in the PrEP-RN service, which collected data related to sex, gender, sexual orientation, ethnicity, and reason for referral. Engagement in PrEP-RN care (including length of enrollment) was tracked in a database as part of program monitoring.

**Table 1:** Interview guide.

Theme	Prompt
***Experience with PrEP-RN***	How/what was your experience accessing PrEP in community-delivered settings? Were things missing from your experience? If yes, what? Was the healthcare staff helpful? Did they answer your questions? Were they sensitive? If no, what was missing?
***Previous Experience(s) using PrEP***	Have you ever taken PrEP before? If yes, was your experience using PrEP-RN different? If yes, what were the differences? If yes, which did experience did you prefer? Have you ever taken PEP before?
***Recommendations for Project Improvement / Development***	What would you recommend to improve PrEP-RN? What other PrEP or HIV prevention strategies would you recommend?

### Data analysis

Applied thematic analysis was completed for all interview transcripts, following the process detailed by Guest et al [17]. For this, all interviews were read and re-read with an emphasis on key aspects related to the research objectives. The focus of this analysis was to examine the ways in which patients constructed the role of the nurse in HIV prevention care—for example, what aspects they found useful, thoughts on the care they received from nurses, identified limitations of nurse-led PrEP and the structure of PrEP-RN, and how their experiences compared to interactions related to PrEP care with other healthcare providers.

As part of this analysis [17], texts were segmented based on thematic elements, including notation of similarities, differences, and overlapping elements within the data. Segmented texts were subsequently identified, and labels were produced which captured the content of each segment [17]. These labels were documented in a codebook. These codes were then clustered into categories based on their overall meaning and their relationship [17] to the role of the nurse in HIV prevention care. Possible themes were noted based on repeated occurrences and events within the data [17]. Major themes were identified [17] by naming the grouped codes based on similarities, differences, and patterns and according to the inferred meaning they attributed to the research objectives, in this case, the patient perception of the role nurses in HIV prevention care.

Separate analyses were completed by each author and were discussed collaboratively to establish the major codes and themes and to ensure consensus of interpretations.

### Ethics

As part of the research study, PrEP-RN patients provided written consent for (1) data to be collected related to their PrEP care and (2) to be contacted for an interview to share their experiences related to PrEP care and accessing nurse-led HIV prevention services. Verbal consent was obtained by the research assistant prior to initiating the interview. Research ethics approval for this study was obtained from the University of Ottawa (H-04-18-533) and Ottawa Public Health (as the organization who operates the Sexual Health Clinic in Ottawa). Research funding for this project was provided by the Ontario HIV Treatment Network.

## Results

The 14 interview participants all identified as gay or bisexual cis-gendered males. The average age of these individuals was 33 years (range: 22–43 years old) and over two thirds reported their ethnicity as white. In terms of reason for referral, 43% (n = 6/14) had accessed the Sexual Health Clinic for PEP. The remaining 57% (n = 8/14) either had a recent STI diagnosis, were named as a contact of HIV, or were referred under nurses’ clinical judgment based on identified risk practices. The average length of enrollment with the PrEP-RN clinic was 7.6 months—with half of all participants being enrolled for the program maximum of 12 months and then being referred to an alternate provider for ongoing PrEP care.

**Fig 1:**
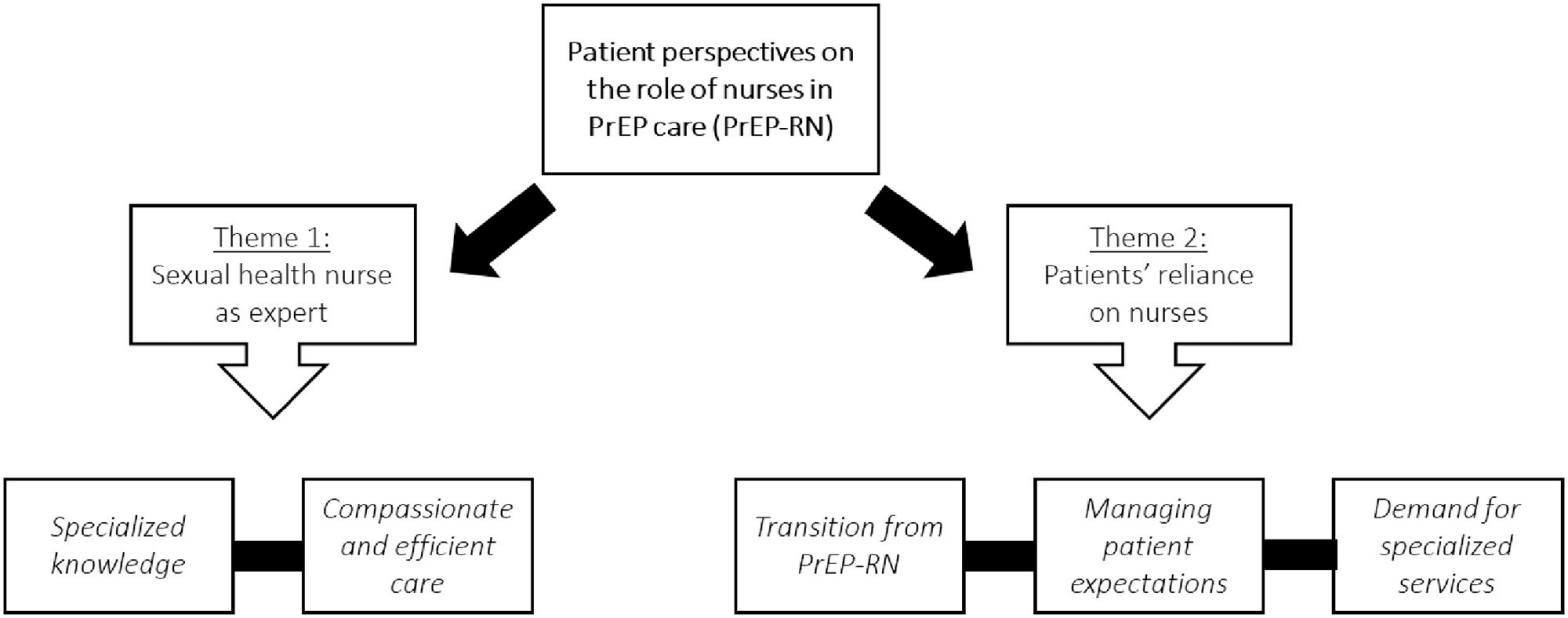
Overview of themes and subthemes.

Analysis from the qualitative interviews yielded two major themes related to the patient perception of the role of the nurse in HIV prevention care: *The Sexual Health Nurse as the ‘Expert’* and *Patients Reliance on Nurses*. These themes were explored with a focus on the construction of the ‘nursing role’–including the implicit and explicit expectations patients held for nurses—as well as the potential implications of patients’ views for nurses working in specialized areas of care, such as sexual health nursing. See Fig 1.

### Theme 1: The sexual health nurse as the ‘Expert’

The first theme focuses on the construction of the nurse as an expert in sexual health care. Within this theme, participants described the quality of care provided to them in terms of nurses’ knowledge and resourcefulness, ability to accommodate individual needs (in ways they felt were different from the approach of other healthcare providers), and the efficiency of the clinical services they received. While described in different ways, participants’ sentiments toward nurses primarily related to the tasks nurses completed related to PrEP care, rather than the value of the nurses’ role.

#### 1.1. Specialized knowledge

Most participants described the sexual health nurses as possessing a high degree of knowledge and skill related to sexual health and PrEP care, which many found comforting as they navigated the process of PrEP initiation and stabilization. Some felt that nurses’ resourcefulness stemmed from their specialized education in sexual health, in terms of the teaching and information nurses provided at visits or their general approach to patient care. For participant 2, the “nuanced training [of nurses] on how to approach people without feeling judged” helped to “establish a sense of safety” during their visits to the Sexual Health Clinic for PrEP. Similar feelings were echoed by other participants, who stated:

*[The nurses are] always very good at providing information and they’re very thorough with their assessments*. *They ask a lot of questions*. *It’s obvious that they care about what’s happening with you and want to make sure you’re leaving with the correct information*

–*P12*

*There has never been a moment where [the nurses] were not knowledgeable or teachable… Most of them have been doing it for long enough that they know how to interact with someone*

–*P13*

*The nurses were wonderful… I really appreciated having access to the resource*. *I didn’t feel judged and they seem knowledgeable and professional*

–*P4*

*Speaking to a nurse—the expert—is just so much easier*.

–*P13*

Other participants felt the expertise of nurses related to their ability to provide clinical services and to monitor test results. Participant 7, who had concerns regarding potential renal effects secondary to PrEP use, described a feeling of reassurance from nurses “looking at my charts and tracking my progress really efficiently”. For this participant, nurses’ intuitive sense of “knowing [their patient] and their history” was a “really important” component of healthcare—particularly when engaging in long-term therapies, such as PrEP.

Similar sentiments were echoed by Participant 14, who had initially presented to the Sexual Health Clinic for PEP and then transitioned to PrEP, who stated: “I like dealing with the same person. It was the same nurse who prescribed me PEP at the beginning, so it was nice to have that journey together”. This consistency in nursing staff was also noted to be beneficial for other participants, in terms of not having to repeat personal stories or having to recount intimate details about their past sexual activities or experiences. For example, Participant 5 shared:

*“There’s a difference between seeing the same [nurse] and having them acknowledge you’ve met before*. *That’s what was beneficial about the clinic*. *The nurses might not remember every detail about me*, *but they would always see me and roughly remember the last time we spoke—which is great when you’re dealing with awkward or difficult healthcare stuff*, *like sexual health*.”

For these individuals, having access to a select group of nurses within the clinic helped them feel supported in their sexual health and PrEP care. This was noted to be a reassuring aspect of care for several individuals, like Participant 2, who once had the experience of being told to “*just be a good boy*” when asking a provider about PrEP, which they felt was “patronizing and homophobic… like I was being told not be a whore”. The approach taken by nurses was useful to maintain relationships with patients who might have experienced stigma or judgements from healthcare providers in the past or who were uncomfortable discussing their sexuality or sexual histories with these other providers. The consistency of nurses’ knowledge and approach thus became an anticipated standard of care for these participants.

#### 1.2. Compassionate and efficient care

In addition to the described expertise of sexual health nurses, many participants spoke of the nurses’ role in terms of the level of compassion they contributed to PrEP care, or the extra measures nurses would often take outside of clinical visits to ensure patients’ needs were met. Some also spoke of the efficiency of services provided by nurses, which meant they did not experience long wait times related to their PrEP care. For these participants, the level of care provided by nurses seemed to exceed what they had experienced in other healthcare settings. On this, participants stated the following:

*The nurses helped me with what I needed*. *Whenever I needed something urgent there*, *I was seen quickly… It was a good resource considering I was a bit worried about everything when I started*.

–*P11*

*They were really supportive*. *For PEP*, *not a lot of stuff was open*, *but they helped me access the medications by connecting me with a certain pharmacy they knew could see me right away*.

–*P14*

*I’m not Canadian born*, *so English is not my first language*. *The way [nurses] explained things to me was very clear and they were willing to help me avoid any other stress from [accessing] PEP and getting started on PrEP*.

–*P9*

*[The nurse] was probably one of the most compassionate people I’ve ever had… which has not always been my story*. *It was definitely important to have somebody show me that they care*.

–*P6*

*I really liked the staff*. *Everybody was gentle and helpful and very accommodating with my phobias of needles*.

–*P1*

The way that nurses accommodated the specific needs of patients during PrEP care was an element participants appreciated—particularly in situations they felt to be “urgent” or when they were experiencing “stress” or “worries” related to their sexual health. Given that nearly half of these participants had been referred to PrEP-RN after having used PEP, the accessibility of nurses to assuage their concerns or assist with linking them to other clinical services (such as a pharmacy) made them feel “cared” for.

In addition to the compassionate care provided by nurses, participants spoke of the structure of the nurse-led PrEPclinic. In the general Sexual Health Clinic, services were previously available on a walk-in basis, meaning presenting to the clinic would not guarantee patients would be seen, while for PrEP-RN, patients were given an appointment. The dedicated hours of operation and consistency of nursing staff, resulted in more streamlined visits, which participants felt eased the process of completing routine PrEP monitoring and STI testing. On this point, they stated:

*It was pretty easy from a patient standpoint*. *Going into the clinic and that experience was always very quick*. *The nurses who were administering it were always efficient and nice*.

–*P5*

*The clinic itself is very well run*. *We got in quickly and did the tests and they manage all of that very well*.

–*P10*

*Some days are slower to get seen [at the clinic] than others*, *but for the PrEP clinic*, *it was only a certain time of day*, *so I usually had an appointment*, *and it was pretty quick to get in and out of*.

–*P3*

Based on these statements, the shift toward appointment-based services in the PrEP-RN clinic appeared to increase participants’ satisfaction with their clinical care. While the level of nursing knowledge or expertise was not different for the PrEP-RN clinic compared to the general sexual health walk-in, the fact that participants did not have to devote a great deal of time to completing PrEP monitoring or STI screening was something they valued—especially because participants were completing follow-up for PrEP every 3 months, and thus, in some cases were, accessing the clinic more frequently than they might have prior to using PrEP. While a seemingly minor detail in the overall structure of care, having dedicated time with PrEP-RN nurses allowed participants to feel acknowledged and respected in their healthcare interactions.

In this theme, the role of *The Sexual Health Nurse as the ‘Expert’* was explored. The statements from participants constructed these nurses as specialists—in terms of the level of knowledge related to sexual health and HIV prevention and nurses’ non-judgmental and flexible approach. Participants reported feeling comfortable to engage in discussions about their sexual health and HIV/STI risks, which gave them reassurance about the quality of healthcare they received. In other words, through expert abilities to interact with patients regarding sensitive topics, nurses could readily identify a comprehensive plan of care—including recommendations for specific testing, vaccinations, or when an assessment by a prescriber (nurse practitioner or physician) might be indicated. The tailored health services nurses provided led participants to feel assured about (1) their overall wellbeing, in that they knew they could access care without feeling stigmatized, and (2) the quality of care they were receiving, in that they could trust the nurses to appropriately identify their needs and manage their follow-up.

### Theme 2: Patients reliance on nurses

As revealed above, participants expressed favourable sentiments toward the knowledge and skills of PrEP-RN nurses. Building on this ‘expert’ construction of nurses, the second theme explores patients’ stated reliance on nurses, created (directly and indirectly) through the specialized care provided by PrEP-RN nurses. Specifically, this theme looks at some of the challenges encountered by participants with respect to their transition in PrEP care from PrEP-RN to primary care provider clinics and some of their identified barriers to accessing health services with these alternate providers. The experiences—and expectations—of participants generated demand for more accessible sexual health and HIV prevention services, something many participants felt could only be accomplished through sexual health clinics and its nurses.

#### 2.1. Transition from PrEP-RN

Patients followed in the PrEP-RN clinic were enrolled for a period of up to 12-months (1) to accommodate new patients who were identified as being at-risk for HIV to have access to rapid PrEP initiation and (2) to build capacity of primary care providers to prescribe PrEP, by referring patients who had already been stabilized on PrEP for ongoing follow-up. At a healthcare systems level, this approach had the advantage of increasing PrEP access to individuals belonging to the groups most affected by HIV, such as gbMSM; however, at a patient-level, the transition of PrEP care from PrEP-RN to primary care settings was not always well received.

Despite being advised of the program structure at the outset, some participants shared concerns about leaving the familiarity and expertise of nurse-led HIV prevention services:

*They told me they will refer me to a doctor [after a year]*, *but I feel more comfortable with this clinic*. *I wish it was longer*, *or just undetermined*.

–*P9*

*I did find [the nurses] very empathetic and easy to work with*. *I just wish I could have stayed with them the whole time*

–*P14*

Other participants noted challenges around the transfer of their medical care and the perceived lack of skills or knowledge specific to PrEP held by alternate care providers:

*I just wish the transition period was just a little smoother*. *My doctor tried to shift me over to the specialist only to do these routine tests*, *but she is my family doctor and I didn’t want to talk to somebody else about it… If each medical facility or specialist could give their colleagues a mandatory training on PrEP*, *it could help reduce stigma and confusion*.

–*P2*

*I decided to go to my family doctors… but then it felt like my doctor didn’t know anything about [PrEP]*. *I had to train her up*.

–*P14*

*The way [care] was transferred to my family doctor could have been a little bit smoother… he did it eventually*, *but he said he needed to do more research first*, *so I had to wait a while*.

–*P6*

Based on these statements, the “ease” and “comfort” of receiving PrEP from nurses in the Sexual Health Clinic led participants to question the care they received elsewhere. While the transition was arranged in advance with primary care clinics, participants became less confident in these providers when “confusion” arose regarding their HIV prevention care. It was not that these physicians lacked the qualifications to provide PrEP. Rather, it appears participants had grown accustomed to accessing services from sexual health providers who could readily respond to items regarding their sexual health and PrEP care, as opposed to general providers who might not be as familiar with HIV prevention guidelines.

#### 2.2. Managing patient expectations

Building on the identified challenges related to the transfer of PrEP care, participants spoke of the differences in services between the Sexual Health Clinic and primary care providers. Several participants expressed some unease about discussing their sexual practices or HIV risks with providers, with Participant 5 noting, “I’ve had family doctors I might not be as eager to have a conversation about my sexual health with”. Additional encounters with primary care providers related to their sexual health needs were described by participants as follows:

*Maybe it’s just me psychologically*, *but I kind of think of my GP as my mom or something and I don’t want her to know about my sexual practices*.

–*P7*

*One of the reasons I’m not a terrible fan of my family doctor is that I get the sense there’s a judgement there that may not exist with heterosexual patients*. *I’m not even sure its something she knows she’s doing*. *It’s just one of those things I pick up on and I’d rather not deal with*. *So*, *this type of clinic is really valuable to get around that type of barrier*.

–*P10*

*It was pretty easy getting PrEP at the sexual health clinic—especially because you could also get all the sexual health testing needed and honestly*, *I don’t really feel comfortable going to my doctor for that*.

–*P6*

In these statements, participants voiced their preference to have their sexual healthcare completed in settings where, and with providers who, they felt were more receptive to discussions about their risk practices. While stigma or judgments was not a universal experience for participants, anticipated discrimination—in terms of “not wanting [providers] to know” about their sexual history or “not feeling comfortable” discussing their risk practices—was noted to be a “barrier” for many to accessing sexual health screening and PrEP care. Notably, participants’ perceptions of primary care providers’ ability to deliver PrEP and STI care seemed to be described in stark contrast compared to PrEP-RN nurses, despite both sets of providers operating under the same standard of care (e.g., testing modalities, clinical guidelines, and practice recommendations).

#### 2.3. Demand for specialized services

Despite these identified barriers, when asked about the ideal setting for HIV prevention care—including PrEP monitoring and STI screening—participants were somewhat divided in their responses. Some, like Participant 13, felt primary care providers were the ideal venue to receive PrEP care because they “will be so much more aware of my history” and “having another specialist step in and have to read over every single file doesn’t make a lot of sense”. For this participant, the connection to a limited number of healthcare providers made them feel more secure, knowing they would not have to disclose their “history” to multiple people.

Other participants echoed similar sentiments around their hope to see PrEP services expanded to primary care clinics in the future. On this, they shared the following:

*Make PrEP more available*! *Just generally for any STI or sexual health related services*, *it needs to be way easier and more accessible for people*

–*P4*

*My experience at the sexual health clinic and being on-boarded to PrEP was very positive*. *I just feel like it needs to be more readily available… Access to this type of care is just so limited*. *It shocks me that there’s no other portals*.

–*P14*

*If I were lucky and got a really cool*, *open-minded family doctor who knew their shit*, *I’d have them do it all for me*. *That would be nice—a one-stop-shop*.

–*P7*

*One thing that I found really annoying is that whenever [a provider] wanted to talk to me about PrEP or sexual health*, *the first thing they told me was to go to the sexual health clinic… There needs to be a mandatory educational workshop that every primary care clinic has to take [because] I’m sure there are gay people who would like to be on PrEP that are using these clinics as the first point of entry*, *and it would be nice if providers were aware of the services available*.

–*P2*

These statements from participants capture a willingness to engage in sexual health services outside of specialized clinics. The primary obstacle for these individuals, however, related to a lack of “accessibility” of providers who were “open-minded” to the intricacies of providing this type of care. Implicit in these statements was the perception of participants’ health needs being dismissed when primary care providers attempted to refer them to other healthcare settings (like the Sexual Health Clinic), rather than provide STI testing or PrEP themselves. In addition, participants identified a need for providers to receive “education” about the resources available locally and how to provide culturally sensitive care when working with gbMSM.

Another subset of participants felt that PrEP and STI services should exclusively be provided by sexual health clinics. The positive experience of receiving care from nurses in settings like PrEP-RN, led participants to feel that specialized clinics and providers were best suited to provide HIV prevention services. This perception builds on previous statements made around the knowledge, skill, and judgement of nurses, which led participants to feel sexual health nurses were the ‘only’ providers who should—or could—administer PrEP services. On the topic of the ideal mode of PrEP service access, participants stated the following:

*I thought [my experience] was wonderful*. *It was who should be providing PrEP in the sense of it’s the sexual health clinic*.

–*P6*

*When I got my family doctor*, *I was very upfront that I was gay and what my sex life was like*. *There wasn’t much of a concern*. *It was more apathy… As good as she is*, *she just doesn’t have the expertise and subject knowledge as someone at the sexual health clinic*.

–*P14*

*You need someone who understands what the [sexual] risks are*. *Kind of an expert on the subject matter who can give you all the information so you can decide for yourself how much risk you’re willing to take*.

–*P8*

Another participant spoke of the potential risks of losing access to this specialized mode of PrEP delivery if patients will not, or cannot, obtain such care through other settings. Given the previously identified barriers to discussing STI and HIV risks with primary care providers, the statement from this participant seems to place the burden of HIV prevention onto sexual health nurses:

*You’ve got people falling through the cracks of the healthcare system who are engaging in possibly risky behaviour that might not even be aware their behaviours are risky…*. *The sexual health centre offers that safety-net*. *So*, *my biggest recommendation would be to keep this pilot project permanently funded within the sexual health clinic*.

–*P2*

Based on the views shared by our participants, it is evident there is strong support among these gbMSM participants for PrEP delivery by nurses. Participants reminisced on the approach of nurses in this setting, who provided “wonderful” care with their “expert knowledge”–not only of PrEP, but also of how to manage sexual risks. From these statements, it seems that nurses’ approach to HIV prevention care extended beyond serologic monitoring and medication delivery. Participants expressed a feeling of nurses safe-guarding multiple aspects of their life by addressing concerns regarding their risk-taking behaviours. For those participants who were amenable to receiving PrEP from primary care physicians, all indicated they would only feel comfortable doing so if speciality trained nurses, like those in PrEP-RN, completed training to physicians. The safety provided through nurses’ expansive list of preventative services became a motivating factor in participants selection of nurse-led PrEP as an ideal model of care.

In summary, this theme explored the *Patients Reliance on Nurses*. Participants described the difficulties they felt when transitioning from the PrEP-RN clinic to primary care providers for ongoing HIV prevention care. Many expressed their preference to continue accessing PrEP from nurses for an undetermined period of time; however, given the structure of the program, participants were referred to alternate providers within 12 months of initiation. As a result of this undesired transition in care, participants recounted differences in the type of care they received from primary providers, making comparisons to the services they had previously received from nurses in the Sexual Health Clinic. The type of care delivered by PrEP-RN nurses may have created elevated expectations for how participants felt PrEP care ought to be provided, which in turn, may have impacted their perspectives on primary care providers.

In discussing the ideal structure and providers for future PrEP care, participants were equivocal in their views. Some felt that PrEP should be more widely accessible in all primary care settings, while others were adamant this only be provided by sexual health nurses. While on the surface, participants appeared to be divided in their thoughts on the future PrEP care, it seems those who were interested in receiving services from primary care providers were only willing to do so if the model of care better emulated that of PrEP-RN. Combined, these sub-themes reveal how, despite the exceptional care provided by nurses, the tendency of these nurses to meet high clinical standards on aspects related to sexual health and PrEP care may have created some unrealistic expectations for participants when receiving care from alternate providers. As a result, these participants may have unintentionally become dependent on nurses’ care.

## Discussion

Herein, we reported on the findings from qualitative interviews completed with gbMSM on their experiences with receiving care in a nurse-led HIV prevention service, known as PrEP-RN. Thematic analysis of these interviews revealed two major themes related to the patient perception of the role of nurses in HIV PrEP delivery, which were, *The Sexual Health Nurse as the ‘Expert’* and *Patients Reliance on Nurses*. Within these two themes, participants expressed their views about how nurses provided exceptional HIV prevention care that was both informative and individually tailored to patients’ unique healthcare needs. The perceived difference in the caliber of HIV prevention care between PrEP-RN and primary care led participants to become distressed when they were referred to alternate clinics for PrEP monitoring. Despite being aware of this transition in advance, participants voiced concerns regarding their new providers, noting the standard of care was not the same as many had become accustomed to receiving from PrEP-RN nurses. Considering that both nurses and physicians were following the same guidelines for PrEP monitoring, it is likely that participants’ misgivings about ongoing care related more to differences in the approach to PrEP follow-up between these two provider groups.

This paper adds to the literature that nurses can provide PrEP safely. In fact, based on the sentiments of our participants, it might even be the preferred delivery model. The specialized knowledge and personalized approach of nurses gave participants a sense of safety both in the care they were receiving (e.g., the abilities of nurses to effectively monitor and administer PrEP), and in the supports they received from nurses (e.g., information, counselling, linkages to care). Indeed, these participants wanted nurses to not only help them with PrEP and other aspects of their life, but also take responsibility for fixing the whole healthcare system for them by providing training to primary care providers on how to provide safe and inclusive PrEP care to gbMSM. As calls for the task-shifting of HIV prevention care to nurses increase [18], the positive insights shared by these participants provide support for nurse-led PrEP, which to date in the literature, has focused on feasibility within the healthcare system and physicians’ interpretations of nurses’ readiness to do so [19, 20]. Thus, in addition to established protocols for nurse-led PrEP (see O’Byrne et al., 2019) [13], having a first-hand account from patients who received PrEP care from nurses could help to advance task-shifting initiatives from a theoretical discussion to a practice reality.

The perception of patients on the role of sexual health nurses in HIV prevention care also raises some interesting points of reflection for nursing practice. For nurses, the impetus of practice is to “work autonomously and in collaboration with others to enable individuals, families, groups, communities, and populations to achieve optimal levels of health” [21 p.5]. As such, nurses’ training is centered around the five-main competency standards of: professional conduct, evidence-based care, ethical practice and decision-making, accountability, and competence [21]. In the capacity of PrEP-RN, nurses entered into a more advanced clinical role, where under medical directives from nurse practitioners, they were required to identify, recommend, and complete necessary testing and clinical services (e.g., vaccinations, STI treatments, etc.), interpret and monitor test results (including serum creatinine), and dispense PrEP prescriptions and medications. This change in practice led to nurses assuming the role of responsible provider for HIV prevention care—much like the role of a primary care provider.

Despite this shift, the core tenants of nursing practice remained an integral component of nurses’ approach to HIV prevention care. Instead of strictly providing the recommended testing and follow-up according to PrEP guidelines [5], nurses incorporated their professional standards and training into their work to ensure patients “achieved optimal levels of health” [21 p.5]–in this case preventing onward risk of HIV acquisition by reducing as many barriers as possible to PrEP care. While patients reported how much they appreciated the nurses’ “knowledge”, “skills”, and “accommodation”, our participants failed to recognize the larger role nurses were playing in HIV prevention care—in terms of nurses’ role as primary providers and the added responsibilities of monitoring PrEP results and administering prescriptions. In turn, patients inadvertently placed pressure onto nurses to address and accommodate multiple competing needs (e.g., risk reduction counselling, obtaining insurance, rearranging appointments, etc.) because they expected nurses to assist them.

This view aligns with socially constructed perceptions of nursing as a ‘helping’ and ‘caring’ profession—where the “essence of nursing practice is the nurse-patient relationship” [22 p.E18]. As such, the role of nurses is often defined by their ability to complete specified tasks and provide supports to patients, families, and caregivers, in times of need. These perceptions of nurses, while longstanding, became a focal image of the COVID-19 pandemic, with mainstream media outlets [22, 23] heralding nurses as “healthcare heroes” for their contributions to healthcare during a time of considerable social panic. Within this “hero” narrative, nurses were portrayed as disciplined subjects, by fulfilling their professional obligations to provide care and assist patients; in return, nurses were awarded social thanks and praise [24, 25].

While the ‘caring’ archetype of nurses has been contested by some nursing researchers and scholars as promoting nursing as a magnanimous profession, among those nurses whose experience is limited to clinical practice, the impetus to assist in need is almost preordained [26, 27]. In taking on the more advanced position of HIV PrEP provider, nurses struggled to delineate their role from an accessory in sexual health care to a fixture in PrEP delivery. Much like the social push nurses faced to be ‘leaders’ in the COVID-19 pandemic by providing ‘exemplary’ patient care with exceptional sacrifice, PrEP-RN nurses faced similar influences from patients to undertake the role of HIV prevention care provider, while also safeguarding patients from HIV and STI acquisition. With access to PrEP care being limited (particularly during lockdowns that occurred during the pandemic), and patients reporting ongoing risks for HIV acquisition, nurses assumed the role of ‘hero’ by continuing to operate PrEP-RN while being acquiescent to the requests of participants to ensure ongoing access to, and engagement in, HIV prevention care. In doing so, however, the expectations these patients placed onto nurses began to mount, where retention in PrEP follow-up became an added responsibility of nurses to fulfill—in addition to PrEP monitoring.

Reflecting on the sentiments of participants regarding nurses’ accommodations during appointments, flexibility regarding missed or late visits, and assistance with covering the cost of PrEP medications for those without insurance, it seems nurses’ tendency to go above and beyond in their provision of care may have been motivated by feelings of moral distress. Psychological frameworks for understanding moral distress posit a relationship between *empathy* and *distress*. Specifically, witnessing patients in vulnerable situations can trigger an emotional response, which, based on their training to assist, leads nurses to engage in specific interventions to reduce the hardship of patients under the pretext of ‘patient care’ [28, 29]. From a more critical stance, however, the actions taken by nurses when empathy is triggered may be motivated by nurses’ desire to reduce their own emotional discomfort [28]. The engagement in health-mediating activities for patients is thus precipitated by consciousness—where the experience of ethical tensions (e.g., awareness of practices that might put a patient at risk for HIV acquisition) leads to feelings of guilt, self-reproach, or remorse [29]. Nurses then, focus their energy on specific interventions to resolve their moral distress [29].

When applied to PrEP-RN, nurses’ awareness of the possible risk to patients’ health associated with HIV, in combination with limited access points for PrEP and a professional ethos to develop meaningful relationships with patients, led nurses to take on additional tasks related to patient care to mediate the emotional response (i.e., distress) they felt during their interactions with patients [29]. In this case, nurses faced internal pressures related to their professional role to (1) maintain patients’ health by preventing HIV acquisition, (2) increase PrEP services, and (3) fulfill their nursing standards by providing highly patient-centric care. At the same time, nurses also experienced external pressures from patients to (1) reduce anxieties related to HIV acquisition—particularly because PrEP-RN patients must meet a high-risk threshold for eligibility, (2) provide teaching and share knowledge related to sexual health and risk reduction, (3) be amenable to personal schedules and individual needs, and (4) provide comprehensive PrEP care.

The various ‘pressure points’ nurses experienced to both fulfill their professional obligations and meet patients’ expectations became a moral trigger. When nurses were unable to meet these needs—or, more aptly, when they were unable to resolve their own feelings of empathy—it created a sense of moral distress, which led them to expend additional time, efforts, and energy related to their role in PrEP-RN. More research is required to understand the perspectives of nurses in HIV prevention care and the impact of task-shifting physician roles onto nurses.

## Limitations of the study

This study is limited by several factors. The first is that our participants all identified as male and as gbMSM, and the majority were white. Considering that current PrEP uptake is higher among gay men compared to other groups at-risk for HIV, the experience of accessing PrEP may have been affected by the high degree of acceptability of this intervention within the gbMSM community. Patient perspectives may have differed had this study been conducted among persons of other genders, sexual orientations, ethnic and/or racial backgrounds. Second, participant interviews occurred during the height of the COVID-19 pandemic when stay-at-home orders were in effect. It is possible that the sentiments participants expressed regarding primary care providers was affected by limited in-person access to PrEP care or the transition to virtual healthcare services. Lastly, while overall retention in the PrEP-RN clinic was monitored as part of study metrics, data were not available on whether participants continued with PrEP after they were referred to alternate clinics. As such, it is difficult to determine if engagement in care is dependent on the healthcare provider type (i.e., are there differences in patient retention rates when PrEP is provided by nurses compared to physicians). Our interpretation of patients’ dependency on nurses for HIV prevention care is thus, likely more of an artefact of specialized providers working in a specialized clinical setting.

## Conclusion

While more research is required to understand the perspective of nurses in HIV prevention care, our interviews with PrEP-RN participants provided some interesting context into the structure and perceived value of healthcare services that are wholly nurse-led. In light of the current constraints to the health system and the shifting of tasks (such as PrEP care) from physicians to nurses, the patient’s perspective is vital in assessing the role and place of nurses within the healthcare system. Overall, our participants reported a high degree of satisfaction with the care they received from sexual health nurses in the PrEP-RN clinic; they felt supported, acknowledged, and safe. Future iterations of nurse-led HIV prevention services should, however, include strategies to ease the transition of patient care to alternate providers, as well as procedures for reducing the burden of care—and moral distress—placed onto nurses.
